# Report of epibenthic macrofauna found from Haima cold seeps and adjacent deep-sea habitats, South China Sea

**DOI:** 10.1007/s42995-020-00073-9

**Published:** 2020-12-09

**Authors:** Dong Dong, Xinzheng Li, Mei Yang, Lin Gong, Yang Li, Jixing Sui, Zhibin Gan, Qi Kou, Ning Xiao, Junlong Zhang

**Affiliations:** 1 Institute of Oceanology, Chinese Academy of Sciences, Qingdao, 266071 China; 2 Center for Ocean Mega-Science, Chinese Academy of Sciences, Qingdao, 266071 China; 3 University of Chinese Academy of Sciences, Beijing, 100049 China; 4 Laboratory for Marine Biology and Biotechnology, Pilot National Laboratory for Marine Science and Technology (Qingdao), Qingdao, 266237 China

**Keywords:** Cold seep, Mud volcano, Ganquan plateau, Epibenthic macroinvertebrates, Faunal composition, South China Sea

## Abstract

This work reports on a preliminary taxonomic study of epibenthic macroinvertebrates collected or observed by underwater video at the Haima cold seeps and in adjacent deep-sea habitats, including a mud volcano field and Ganquan Plateau, during an expedition in the South China Sea by the Chinese-manned submersible *Shenhai Yongshi* in May 2018. A total of 41 species belonging to 6 phyla were identified, among which 34 species were collected from the Haima cold seeps. Mollusks and crustaceans that are specialized in reducing habitats were predominant in biotopes of the Haima cold seeps, whereas sponges and cold-water corals and their commensals were prominent in communities of the mud volcano field and the slopes of Ganquan Plateau. The distribution and faunal composition of each taxonomic group are discussed.

## Introduction

Cold seeps are areas of the seafloor, where hydrocarbon-rich fluid and gases leak from fissures and emerge through the sediments and into the water column, creating unique habitats. Such seepage was first discovered on the Florida Escarpment in the Gulf of Mexico (Paull et al. 1984). Since then, hundreds of cold-seep sites have been discovered and observed globally (e.g., Feng et al. 2018; German et al. 2011; Suess 2014), unveiling a specialist seepage macrofauna. Cold-seep macrofauna, being sustained by chemosynthetic primary production, typically consists of a high abundance of symbiotrophic organisms (Barry et al. 1996; Carney 1994; Levin 2005; Sibuet and Olu 1998; Washburn et al. 2018). Hence, cold seeps exhibit a community structure that is distinct from that seen in the surrounding seafloor environment. During the past three decades, numerous studies have been performed, driven by efforts to explore these special habitats and their associated organisms. Some studies have focused on the taxonomy and phylogeny of organisms associated with cold seeps, aiming to discover new species and new distribution records, evaluate phylogenetic relationships, and reconstruct the origins and evolutionary histories of seepage faunas (e.g., Chen et al. 2018; Dong and Li 2015; Xu et al. 2019). Other studies have focused on the community structures and temporal dynamics of cold seeps, analyzing the correlation between pattern of biodiversity in the infaunal assemblages and environmental factors such as depth and seepage types (e.g., Bourque et al. 2017; Cunha et al. 2013; Levin et al. 2015; Washburn et al. 2018).

The South China Sea is a marginal sea in the western Pacific Ocean with passive continental margins in the west and north, where various cold-seep sites have been discovered, in particular on the continental slope (Fang et al. 2019; Feng and Chen 2015; Li 2015, 2017; Niu et al. 2017). The first active seepage site to be discovered in the South China Sea is known as Site F (also called Jiaolong Seep No. 1, Formosa Ridge or Taixinan cold seep), located in the northeastern region (Lin et al. 2007). In the summer (June–July) of 2013, the corresponding author (Xinzheng Li) participated in the first cruise with experimental applications of the Chinese-manned submersible “*Jiaolong*” in the South China Sea (China Ocean Voyage No. 31). The epibenthic community at Site F was described as being dominated by the alvinocaridid shrimp *Alvinocaris longirostris* Kikuchi & Ohta, 1995, the mytilid mollusk *Gigantidas platifrons* (Hashimoto & Okutani, 1994), the galatheid squat lobster *Shinkaia crosnieri* Baba & Williams, 1998, and other squat lobster and mollusk species (Li 2017). From 2013 to 2018, various Chinese research institutions carried out a series of surveys at this site and gathered numerous macrobenthic specimens using remote operated vehicles (ROV) and the manned submersible *Jiaolong*. To date, 33 epibenthic macroinvertebrate species have been reported from Site F and its rim area, six of which are new to science, revealing high biodiversity and most likely a high level of species endemism (Chan and Komai 2017; Dong and Li 2015; Gong et al. 2015; Li 2017; Sha 2019; Zhang et al. 2016; Zhang and Zhang 2017).

In 2013, a new active cold-seep site was discovered on Four-Way Closure Ridge, not far from Site F (Klaucke et al. 2015). In 2015, another active cold-seep field in the South China Sea was identified on the northwestern continental slope, characterized by patches of authigenic carbonate rocks protruding from the muddy seafloor, which contains at least two seepage sites (Fang et al. 2019; Liang et al. 2017). This area was named the Haima cold seeps and has attracted increasing attentions since its discovery. To date, four new bivalve species have been described based on specimens collected through surveys made at Haima in the past four years (Chen et al. 2018; Jiang et al. 2019; Xu et al. 2019). However, information on other taxonomic groups and community structure found in this field is scarce.

**Fig. 1:**
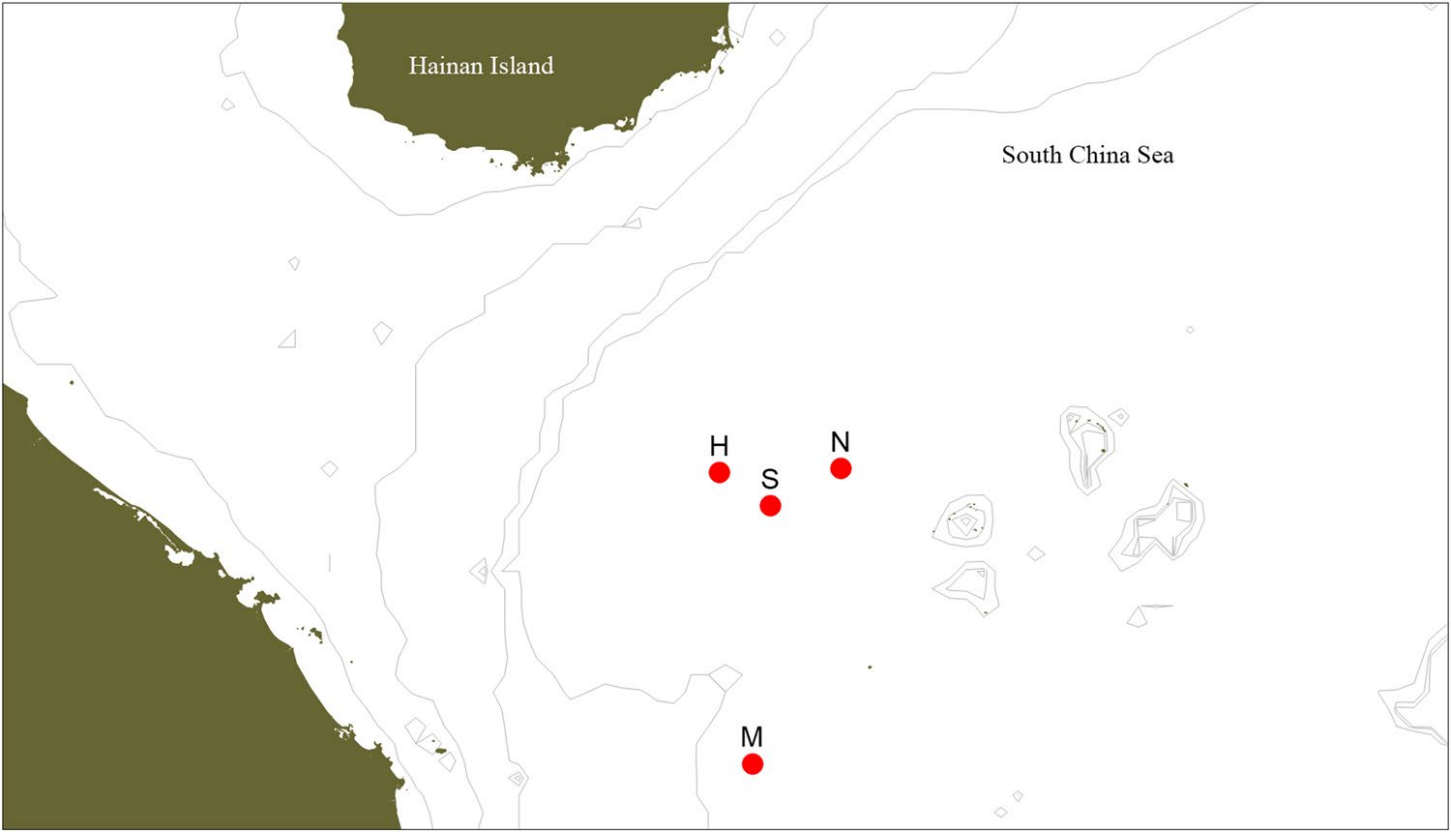
Location map of the study area in the South China Sea. *H* Haima cold-seep field; *N* northeastern slope of Ganquan Plateau; *M* mud volcano field, *S* southwestern slope of Ganquan Plateau

**Fig. 2:**
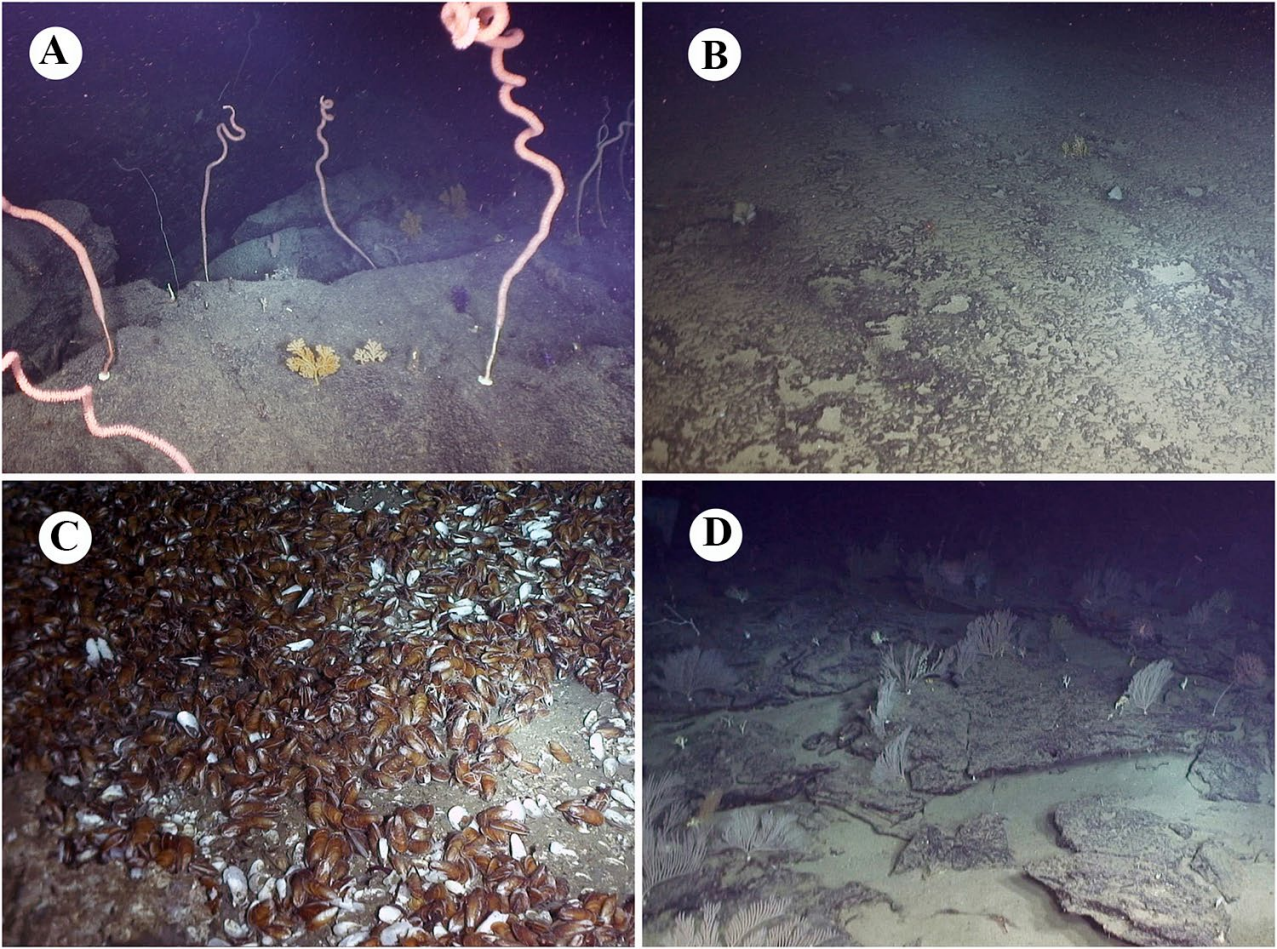
Haibitats investigated during the expedition. **a** Southwestern slope of Ganquan Plateau; **b** northeastern slope of Ganquan Plateau; **c** Haima cold-seep site; **d** mud volcano field

The expedition (Fig. 1) to the Haima cold seeps conducted in May 2018 was organized by Tongji University and Chinese Academy of Sciences. Additional cold-seep sites were discovered, including one on the southwestern slope of Ganquan Plateau, close to the Haima field. The expedition also explored an adjacent mud volcano field, and the slopes of Ganquan Plateau (Fig. 2). The corresponding author (Xinzheng Li) took part in the expedition and dived with the submersible “*Shenhai Yongshi*” (*Deep-Sea Warrior*) at the Haima cold-seep sites. Abundant epibenthic macroinvertebrate specimens were collected or observed in situ using the manned submersible. Some stalked barnacles were subsequently described by Gan and Li (2019). The present study summarizes and reports the taxonomic findings on epibenthic macrofauna from Haima cold seeps and nearby ecosystems, based on specimens collected and photographed during the expedition, to provide an overview of the species composition and biodiversity characteristics of the macrofaunal assemblages in these deep-sea habitats.

## Results and discussion

### Faunal composition and species list

**Fig. 3:**
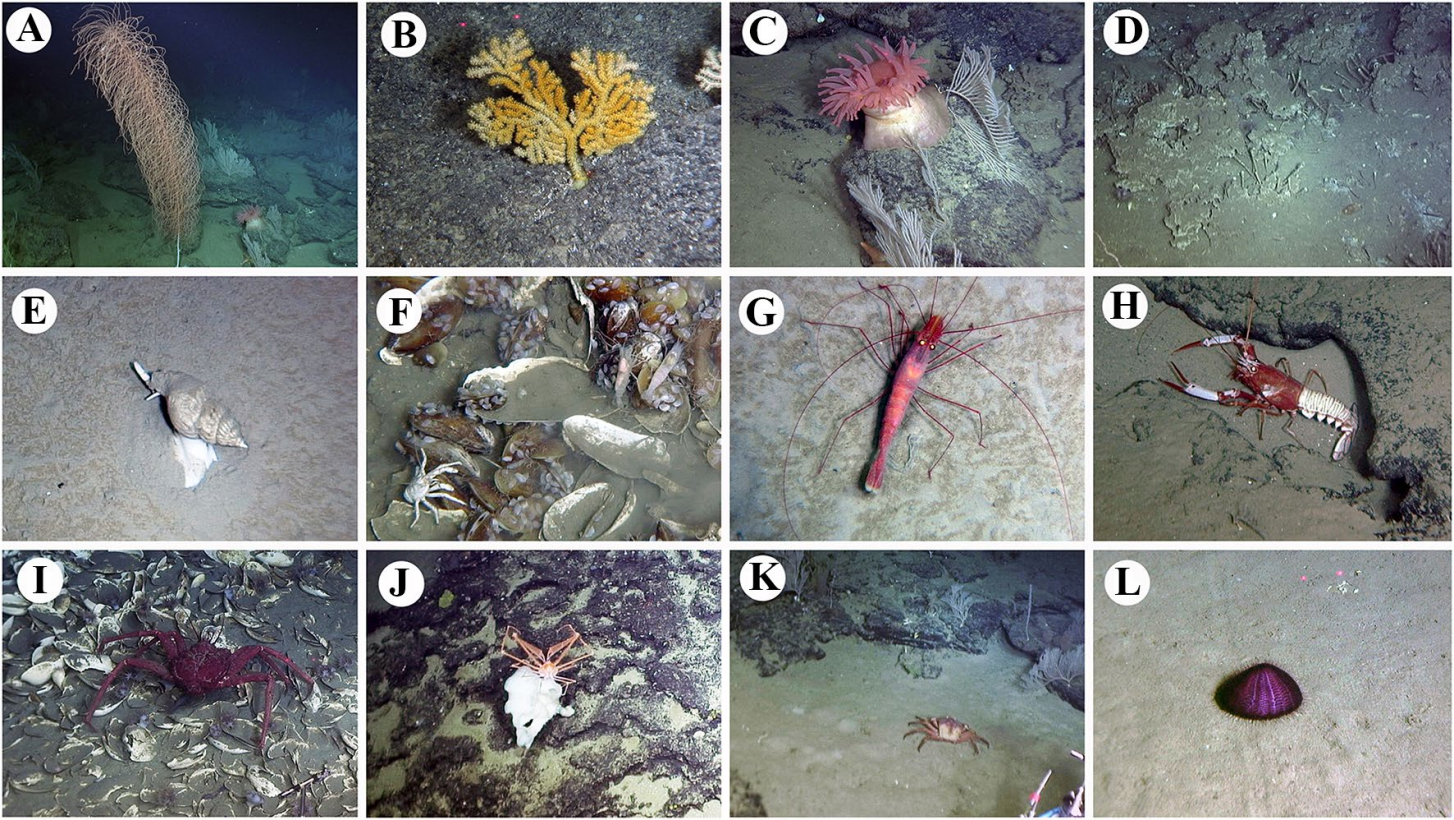
In situ photographs of epibenthic macrofauna taken during the expedition. **a** *Rhodaniridogorgia* sp., mud volcano field; **b** Acanthogorgiidae gen. et sp. indet., southwestern slope of Ganquan Plateau; **c** Actinostolidae gen. et sp. indet., mud volcano field; **d** *Paraescarpia echinospica*, Haima cold-seep site; **e** *Plicifusus* sp., on muddy seafloor around Haima cold-seep site; **f** *Alvinocaris longirostris*) and squat lobster (probably *Munidopsis lauensis*), on mussel bed in Haima cold-seep site; **g** *Nematocarcinus* sp., on muddy seafloor around Haima cold-seep site; **h** *Metanephrops neptunus*, mud volcano field; **i** *Paralomis* sp., in rim of mussel bed in Haima cold-seep site; **j**, *Cyrtomaia* sp., on sponge *Chonelasma* sp., northeastern slope of Ganquan Plateau; **k** *Chaceon* sp., mud volcano field; **l** Echinothuriidae gen. et sp. indet., on muddy seafloor around Haima cold-seep site

**Fig. 4:**
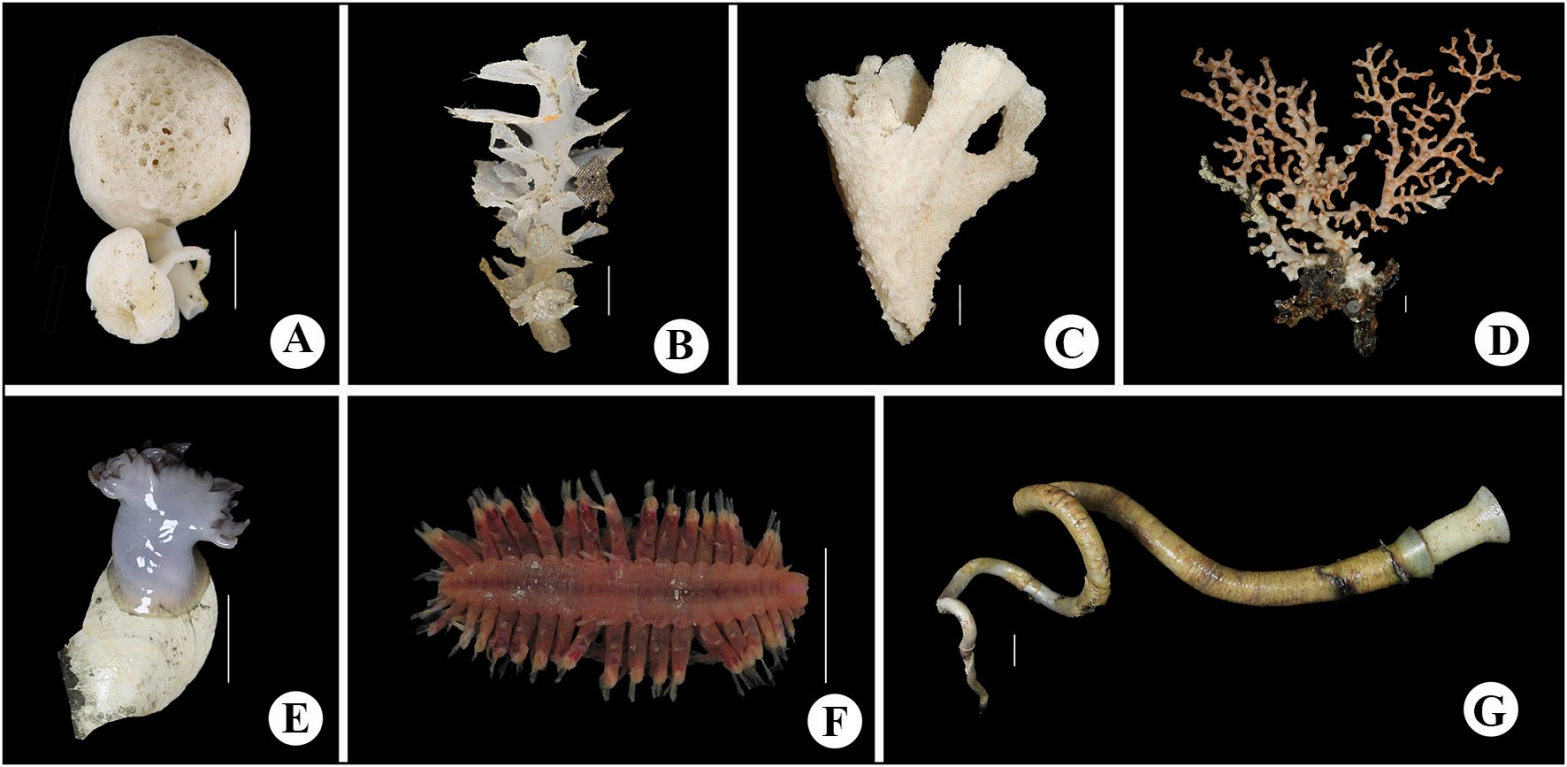
Colour images of freshly collected specimens. **a** *Caulophacus* sp.;** b** *Pleurochorium* sp.; **c** *Chonelasma* sp.; **d** *Enallopsammia rostrata*; **e** *Actinernus* sp.; **f** *Branchipolynoe pettiboneae*; **g** *Paraescarpia echinospica*. Scale bar = 5 cm (**a**,** c**,** e**); 1 cm (**b**, **d**, **f**, **g**)

**Fig. 5:**
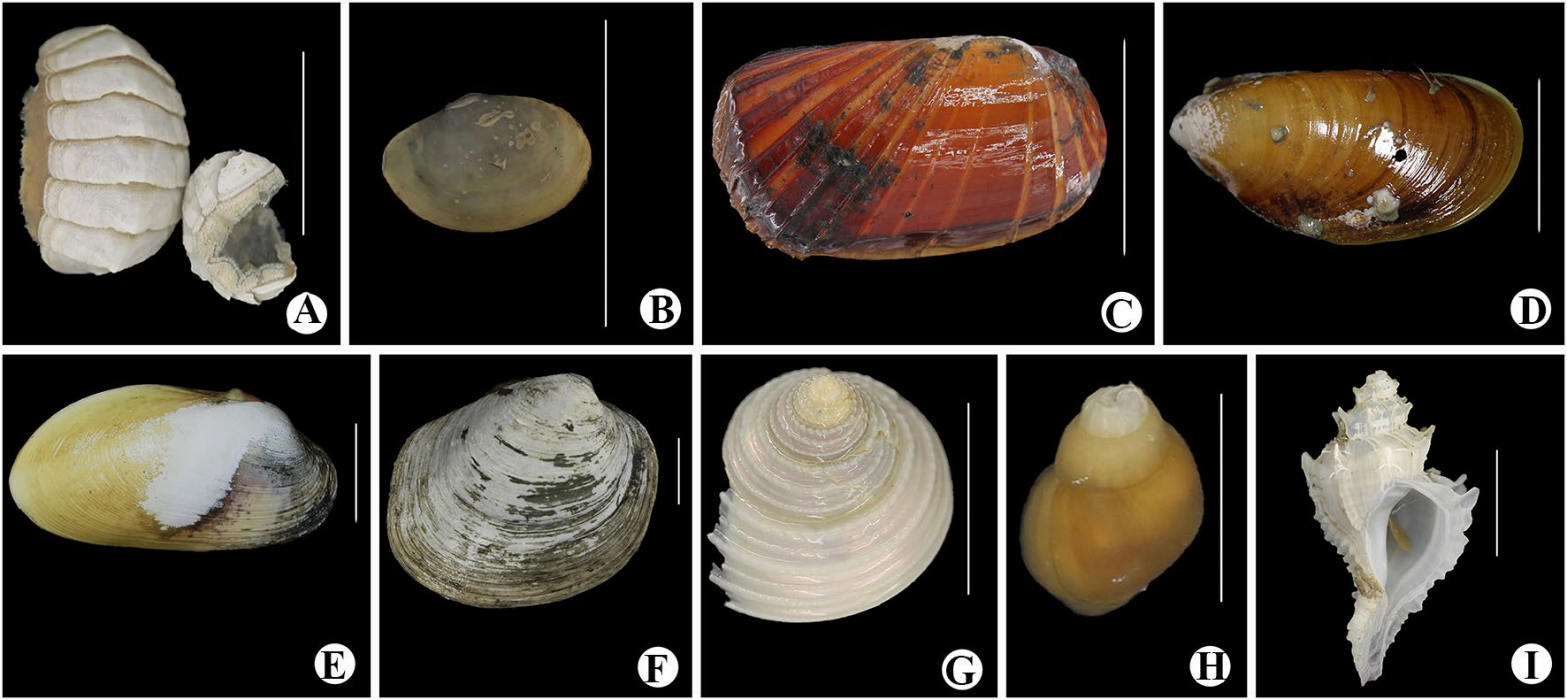
Colour images of freshly collected specimens. **a** *Leptochiton tenuidontus*; **b** *Malletia* sp.; **c** *Solemya* sp.; **d** *Gigantidas haimaensis*;** e** *Calyptogena marissinica*;** f** *Lucinoma* sp.;** g** *Tristichotrochus ikukoae*;** h** *Provanna glabra*; **i** *Scabrotrophon scitulus*. Scale bar = 1 cm (**a**,** b**,** f**,** g**,** h**,** i**); 5 cm (**c**,** d**,** e**)

**Fig. 6:**
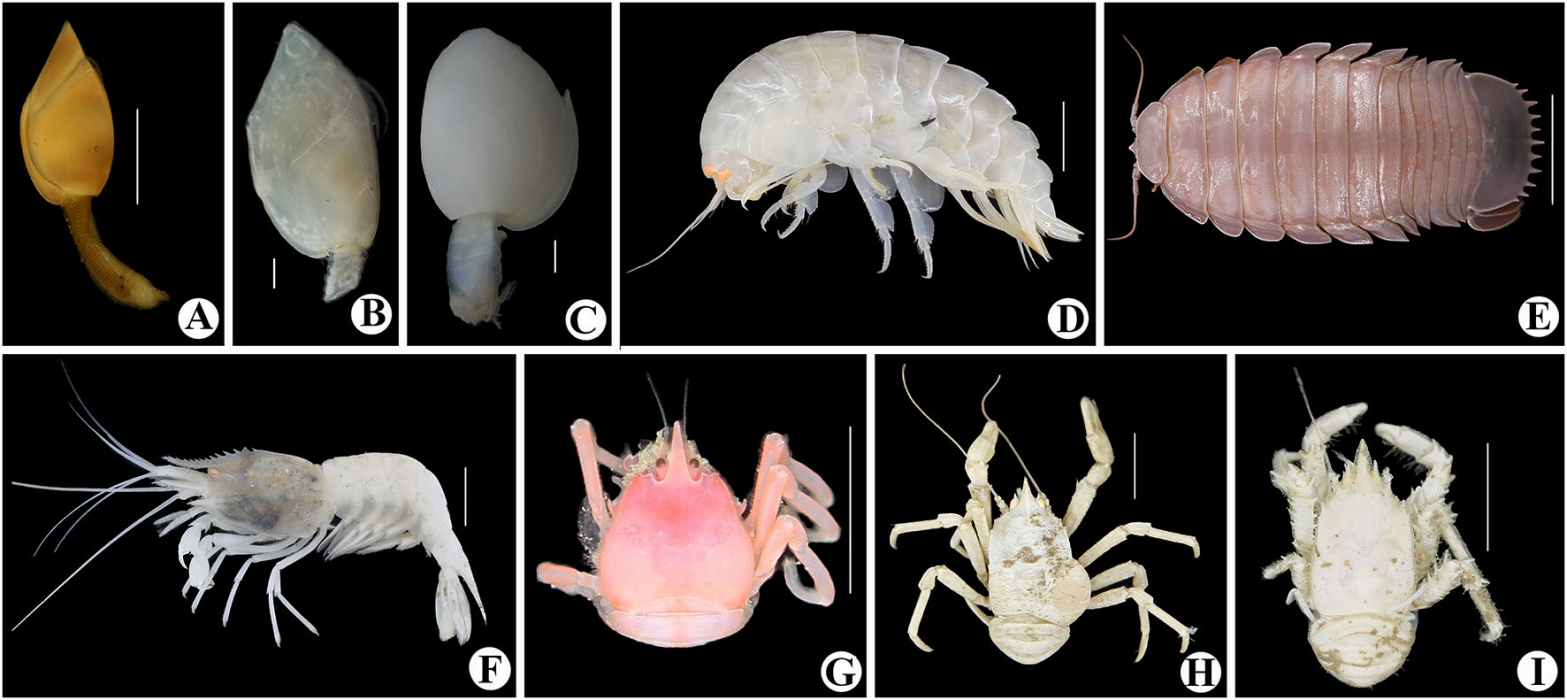
Colour images of freshly collected specimens. **a** *Glyptelasma gigas*;** b** *Poecilasma litum*;** c** *Poecilasma obliqua*;** d** *Eurythenes maldoror*;** e** *Bathynomus jamesi*;** f** *Alvinocaris longirostris*;** g** *Uroptychus setosidigitalis*;** h** *Munidopsis lauensis*; **i** *Munidopsis pilosa*. Scale bar = 5 mm (**a**); 1 mm (**b**,** c**); 1 cm (**d**, **f**, **g**, **h**, **i**); 5 cm (**e**)

A total of 41 species (Table 1) were identified to genus or species level from specimens either collected or observed in situ during the expedition to the slopes of Ganquan Plateau, the Haima cold-seep sites, and a mud volcano field. These comprise 16 species of Crustacea; 14 Mollusca; 4 Cnidaria; 3 Porifera; 3 Annelida and 1 Echinodermata (Figs. 3, 4, 5 and 6). Some other specimens that are pending identification may include undescribed taxa and therefore are not listed or reported here.

**Table 1:** Checklist of the species currently identified to genus or species levels collected from deep-sea habitats of Haima cold seeps, Ganquan Plateau and a mud volcano field in May of 2018

Phylum	Class	Order	Family	Species	Location and habitat^b^	Figure	Remarks
PORIFERA	Hexactinellida	Lyssacinosida	Rossellidae	*Caulophacus* sp.	GP1	4a	Sessile living on rocks
		Sceptrulophora	Euretidae	*Pleurochorium* sp.	GP1	4b	Sessile living on rocks
				*Chonelasma* sp.	GP2	4c	Sessile living on rocks
CNIDARIA	Anthozoa	Scleractinia	Dendrophylliidae	*Enallopsammia rostrata* (Pourtalès, 1878)	GP1	4d	Sessile living on rocks
		Alcyonacea	Chrysogorgiidae	*Rhodaniridogorgia* sp.	MV	3a	Sessile living on rocks
				*Psedochrysogorgia* sp.	GP2		Commensal with Euryalid ophiuroid
		Actiniaria	Actinernidae	*Actinernus* sp.	HM	4e	Adherent to dead shells, especially abundant in the rim area of mussel beds
ANNELIDA	Polychaeta	Phyllodocida	Polynoidae	*Branchipolynoe pettiboneae* Miura & Hashimoto, 1991	HM	4f	Commensal, living within shells of mussels
			Glyceridae	*Glycera* sp.	GP2		Found in crevice of a Pheronematidae sponge
		Sabellida	Siboglinidae	*Paraescarpia echinospica* Southward et al., 2002	GP1, HM	3d, 4g	Usually clusters on edges of authigenic carbonate rocks and occasionally forms small assemblages on mussel bed
MOLLUSCA	Polyplacophora	Lepidopleurida	Leptochitonidae	*Leptochiton tenuidontus* Saito & Okutani, 1990	HM	5a	Among mussles and ophiactids in mussel bed of Haima cold seeps
	Bivalvia	Nuculanida	Malletiidae	*Malletia* sp.	HM	5b	Within mussel bed of cold seeps
		Solemyida	Solemyidae	*Solemya* sp.	HM	5c	Within mussel bed of cold seeps
		Mytilida	Mytilidae	*Gigantidas haimaensis* Xu, Feng, Tao & Qiu, 2019	HM	5d	This species is a chemosynthetic-special mussel, and is one of the most dominant species in the mussel beds of Haima cold seeps
		Pectinida	Propeamussiidae	*Propeamussium* sp.	MV, HM		Identified based on a small colorless specimen associated with dead sponge in mud volcano field. Plenty of *Propeamussium* scallops were also observed free-living upon mud in rim area of mussel beds
		Venerida	Vesicomyidae	*Calyptogena marissinica* Chen, Okutani, Liang & Qiu, 2018	HM	5e	Very common in mussel-bed assemblages
		Lucinida	Lucinidae	*Lucinoma* sp.		5f	Within mussel bed of cold seeps
	Gastropoda	Patellogastropoda^a^	Pectinodontidae	*Bathyacmaea lactea* Zhang et al., 2016	HM		Small in size and predominant in abundance in mussel-bed assemblages
		Trochida	Calliostomatidae	*Tristichotrochus ikukoae* (Sakurai, 1994)	GP1	5g	Identified based on a small specimen associated with a Hexasterophora sponge
			Margaritidae	*Margarites* sp.	HM		Within mussel bed of cold seeps
		Caenogastropoda^a^	Provannidae	*Provanna glabra* Okutani, Tsuchida & Fujikura, 1992	HM	5h	Within mussel bed of cold seeps
		Neogastropoda	Raphitomidae	*Phymorhynchus buccinoides* Okutani, Fujikura & Sasaki, 1993	HM		Within mussel bed of cold seeps
			Buccinidae	*Plicifusus* sp.	HM	3e	Outside of the mussel bed. The species, based on a large specimen (> 9 cm in length), was observed creeping on muddy seafloor in rim of a mussel bed
			Muricidae	*Scabrotrophon scitulus *(Dall, 1891)	HM	5i	Within mussel bed of cold seeps
ARTHROPODA	Hexanauplia	Lepadiformes	Poecilasmatidae	*Glyptelasma gigas* (Annandale, 1916)	MV	6a	Adherent to Isididae coral
				*Poecilasma litum* Pilsbry, 1907	MV	6b	Adherent to carapace of *Metanephrops neptunus *(Bruce, 1965)
				*Poecilasma obliqua* Hoek, 1907	MV	6c	Adherent to the 3rd maxilliped of *Metanephrops neptunus*
	Malacostraca	Amphipoda	Eurytheneidae	*Eurythenes maldoror* d'Udekem d'Acoz & Havermans, 2015	HM	6d	Captured by trapping cage deployed on the seafloor between two seep sites. The cage was put during dive of the day ahead, and collected during the dive of the next day by the corresponding author (Xinzheng Li)
		Isopoda	Cirolanidae	*Bathynomus jamesi* Kou, Chen & Li, 2017	HM	6e	Captured together with *Eurythenes maldoror* using trapping cage by corresponding author (Xinzheng Li)
		Decapoda	Alvinocarididae	*Alvinocaris longirostris* Kikuchi & Ohta, 1995	HM	3f, 6**f**	One of the dominant crustaceans in West Pacific chemosynthetic ecosystems. This species was observed in swarms within seepage mussel beds
			Nematocarcinidae	*Nematocarcinus* sp.	HM	3g	Identified based on an uncollected individual; inhabiting on muddy seafloor in rim of the mussel bed
			Palaemonidae	*Palaemonella* sp.	GP2		Observed in high abundance suspending upon the seafloor
			Nephropidae	*Metanephrops neptunus *(Bruce, 1965)	MV	3h	Common in mud volcano area, inhabiting under stones or in gas holes. Stalked barnacles were recovered from a specimen of *Metanephrops neptunus*
			Lithodidae	*Paralomis* sp.	HM	3i	Identified based on a specimen captured using the submersible’s mechanic arm; in situ observed crawling in the rim area of mussel bed
			Chirostylidae	*Uroptychus setosidigitalis* Baba, 1977	GP2	6g	Associated with *Psedochrysogorgia* corals. The specimen, with the chelipeds lost, was temporarily assigned to *U. setosidigitalis*, although its rostrum was relatively longer than that of the holotype
			Munidopsidae	*Munidopsis lauensis* Baba & Saint Laurent, 1992	HM	6h	Typical species in chemosynthetic habitats, widely distributed in West-Pacific chemosynthetic environment
				*Munidopsis pilosa* Henderson, 1885	HM	6i	Widely distributed in Indo-West Pacific. In the deep waters of Taiwan Island, it was found associated with sunken wood (Baba et al. 2009). This is the first discovery of the species from cold-seep environment
				*Munidopsis trifida* Henderson, 1885	GP1		Widely distributed across Pacific and Indian Oceans. The specimen collected from Ganquan Plateau has been parasitized by an isopod species in gill chamber
			Inachidae	*Cyrtomaia* sp.	GP2, MV	3j	The specimen in northeastern slope of Ganquan Plateau was captured upon a *Chonelasma* sponge. Another small specimen was observed inhabiting on a reefs in mud volcano area
			Geryonidae	*Chaceon* sp.	MV	3k	Common in chemosynthetic environment, which is seen as an opportunist in this kind of habitat. The species was observed crawling on sea bottom in the mud volcano field
ECHINODERMATA	Ophiuroidea	Amphilepidida	Ophiothamnidae	*Histampica* sp.	HM		Inhabiting in mussel bed with other ophiuroids species

^a^Subclass

^b^Location and habitat: GP1: slope of southwestern Ganquan Plateau, depth 1300–1412 m, with the substrates basically boulders and flat rocks, and the only exception of a cold-seep site where the tubeworm *P. echinospica* were collected, GP2: rocky slope of northeastern Ganquan Plateau, depth 586–910 m, with the substrate flat rocks, HM: within mussels bed of Haima cold-seep sites, depth 1380–1390 m, with the substrate muddy seafloor, MV: mud volcano area, depth 500–810 m, with the substrates flat rocks on the otherwise muddy seafloor

The investigation area of the southwestern slope of Ganquan Plateau has a variety of microhabitats. The substrate basically comprised boulders on steep slopes and flat rock on gentle slopes. In addition, a small cold seep at the foot of the plateau was discovered for the first time. Six macrofaunal species were identified based on the specimens collected from this area, including two species of sponges (Fig. 4a, b), one species of cold-water coral (Fig. 4d), and one gastropod species (Fig. 5g) which was associated with the sponge. *Paraescarpia echinospica*, a symbiotrophic tubeworm, was the only organism observed in the small cold-seep site within this study area.

The investigation area of the northeastern slope of Ganquan Plateau is characterized by flat and rocky slopes extending across a large area. Six macrofaunal species were identified in this habitat, including two sessile organisms (a *Chonelasma* sponge (Fig. 4c) and a *Pseudochrysogorgia* cold-water coral), a squat lobster of the genus *Uroptychus* (Fig. 6g) that was associated with the cold-water coral, and a *Glycera* polychaete associated with an unidentified sponge. A *Cyrtomaia* crab (Fig. 3j) was collected from an unidentified sponge, but nature of their relationship is not clear. One species of *Palaemonella* shrimp was observed in high abundance on the sea bottom.

Five cold-seep sites were surveyed during the expedition. Large areas of mussel beds (dominated by *Gigantidas haimaensis*) were observed at these seeps, which is a typical feature of an active seepage ecosystem (Feng et al. 2018). In contrast, patches of relatively small authigenic carbonate hardgrounds were common around these sites, indicating an early stage of ecological succession in some places (Feng et al. 2018). In total, 24 species were identified from the specimens collected at Haima cold seeps, and most were mollusks (13 species) (Figs. 3e, 5a–f, h, i) and crustaceans (seven species) (Figs. 3f, g, i, 6d–f, h, i). The large vesicomyid clam species *Calyptogena marissinica*, which was described first time by Chen et al. (2018) based on samples from the Haima cold seeps, was found and collected again by the corresponding author (Xinzheng Li) in the expedition of 2018. It is a very common species in the cold-seep areas. A red bloody fluid flows from freshly collected specimens of *C. marissinica*. This appearance is very similar to other species of the genus *Calyptogena* (Fujikura et al. 2012). The amphipod *Eurythenes maldoror* (Fig. 6d) and the isopod *Bathynomus jamesi* (Fig. 6e), both of which are opportunistic scavengers, were captured in cage traps. No coral species were observed in this habitat. The symbiotrophic tubeworm *Paraescarpia echinospica*, (Figs. 3d, 4g) occurred in small clusters surrounding authigenic carbonate rocks. The scaled polychaete *Branchipolynoe pettiboneae* (Fig. 4f) and the sea anemone *Actinernus* sp. (Fig. 4e) were very common within and around the cold-seep mussel beds.

A mud volcano field was newly found during the expedition, and its adjacent seabed was investigated. Here, a large area of flat rocks was present on the otherwise muddy seafloor, providing a hard substrate that harbored abundant corals. Eight species were identified based on faunal collections at this mud volcano field. One cold-water coral, *Rhodaniridogorgia* sp. (Fig. 3a), was identified from video images. Six crustacean species, including three species of stalked barnacle (Fig. 6a–c), were either collected or observed by video. A scallop bivalve of the genus *Propeamussium* was the only mollusk observed in this habitat.

### Diversity and distributions of the major macrofaunal groups

Nearly all the sponges observed and collected during the expedition were distributed on rocky slopes of the Ganquan Plateau, whereas no sponges were found at the Haima cold seeps. Similarly, all cold-water corals were collected or observed on the slopes of Ganquan Plateau or at the mud volcano field, where the seafloor was largely composed of boulders or continuous flat reefs, respectively. This is in line with the sessile lifestyle of these two animal groups. Two anemone species were observed in the cold-seep habitat: *Actinernus* sp., which was present in high abundance attached to empty shells, and a species of Hormathiidae, which was found on the seafloor adjacent to one of the seepage site.

Most of the mollusk and polychaete species were found living in the seepage area, where the seabed was mainly mud sediment, providing an ideal habitat for mud-preferring mollusks and polychaetes. High chemosynthetic primary production in cold-seep sites sustains a flourishing seepage community in which chenosynthetic-specialist mussels predominate with high biodiversity and abundance, typically forming large areas of mussel beds. At least eight chenosynthetic-specialist mollusks or polychaetes were collected during the expedition. Only two gastropod species, namely *Tristichotrochus ikukoae* and a species of Cancellariidae, were present on the slope of Ganquan Plateau, associated with a sponge and a cold-water coral, respectively.

Crusateans generally have a strong ability to move, which allows them to adapt to various deep-sea habitats. A high biodiversity of crustaceans was observed in this expedition. However, few species were shared among the four types of habitat, each of which had a distinct crustacean fauna. The rocky slopes of Ganquan Plateau supported plentiful gorgonians; coral-associated squat lobsters, such as species of *Uroptychus* and *Sternostylus*, were also very common. Additionally, the more complicated topographical environment attracted crabs which could shelter and breed under rocks and in gaps. The rocky substrate in the mud volcano area also provides a habitat for crabs and coral-associated crustaceans; solid rocks on an otherwise muddy seabed provided habitat for hole-dwelling species, such as the crayfish *Metanephrops neptunus*. By contrast, the cold-seep sites were colonized by species of squat lobster and alvinocaridid shrimp that are specialists in reducing habitats. Examples include *Munidopsis lauensis* and *Alvinocaris longirostris*, which are generally dominant in cold-seep communities. King crabs, acting as vagrant predators, were also very common in the cold-seep communities.

Members of the Echinodermata constituted an important part of the overall faunal biodiversity of the Haima cold-seep areas. However, the taxonomic identity of these animals was difficult to determine and only one species could be identified to genus level. Ophiuroids (brittle stars) were the most diverse subgroup and were observed in high abundance in the cold-seep assemblages. They included species of *Histampica*, Ophiacanthidae, and Amphiuridae. Conversely, species of Euryalidae were associated with gorgonians at Ganquan Plateau, and some species of Ophionereididae, Ophiuridae and Ophiacanthidae were associated with sponges. A species of sea cucumber, probably belonging to the Chiridotidae, was common in and around cold-seep sites. Sea urchins were scarcely observed, but one large-sized individual representing a species of Echinothuriidae (Fig. 3l) was captured on the muddy seabed beside a mussel bed.

### Preliminary description of community structures and faunal comparison

The community structure of a cold-seep habitat can be influenced by many factors, such as depth, seepage type, and gas composition. Many studies have focused on endobenthic fauna assemblages (micro- and meiobenthos) to quantitatively evaluate their communities in different cold-seep habitats (e.g., Bourque et al. 2017; Cunha et al. 2013; Levin et al. 2015; Washburn et al. 2018).

Although nearly half of the taxa reported here have not been identified to genus or species level, this investigation lends preliminary insights into the community structures of the different types of habitat explored during the expedition. Accordingly, the Ganquan Plateau slopes, characterized by hard substrates, were colonized by high numbers of cold-water corals and sponges along with their associated crustaceans and ophiuroids, revealing faunal community structures similar to those at seamounts. To a large extent, it was the abundance and biodiversity of sessile invertebrates, rather than motile animals, determined the overall epibenthic community structure in these rocky habitats. However, the southwestern slope of Ganquan Plateau, which is deeper and nearer to the Haima cold seeps than the northeastern slope, had a cold-seep microhabitat site; therefore seep-associated faunal species, such as the tubeworm *Paraescarpia echinospica*, were present. The Haima cold-seep faunal assemblages on the muddy seabed revealed typical seepage communities, composed of species that are specialized for life in reducing habitats and opportunistic predators but devoid of cold-water corals and sponges. The mud volcano field, characterized by flat reefs on an otherwise muddy seafloor, shares some community characteristics with the Ganquan Plateau slopes. However, no seep-associated organism was observed in the mud volcano field, probably because the gas release there is less intensive than that in cold-seep sites and thus does not support a chemosynthesis-based assemblage.

**Fig. 7:**
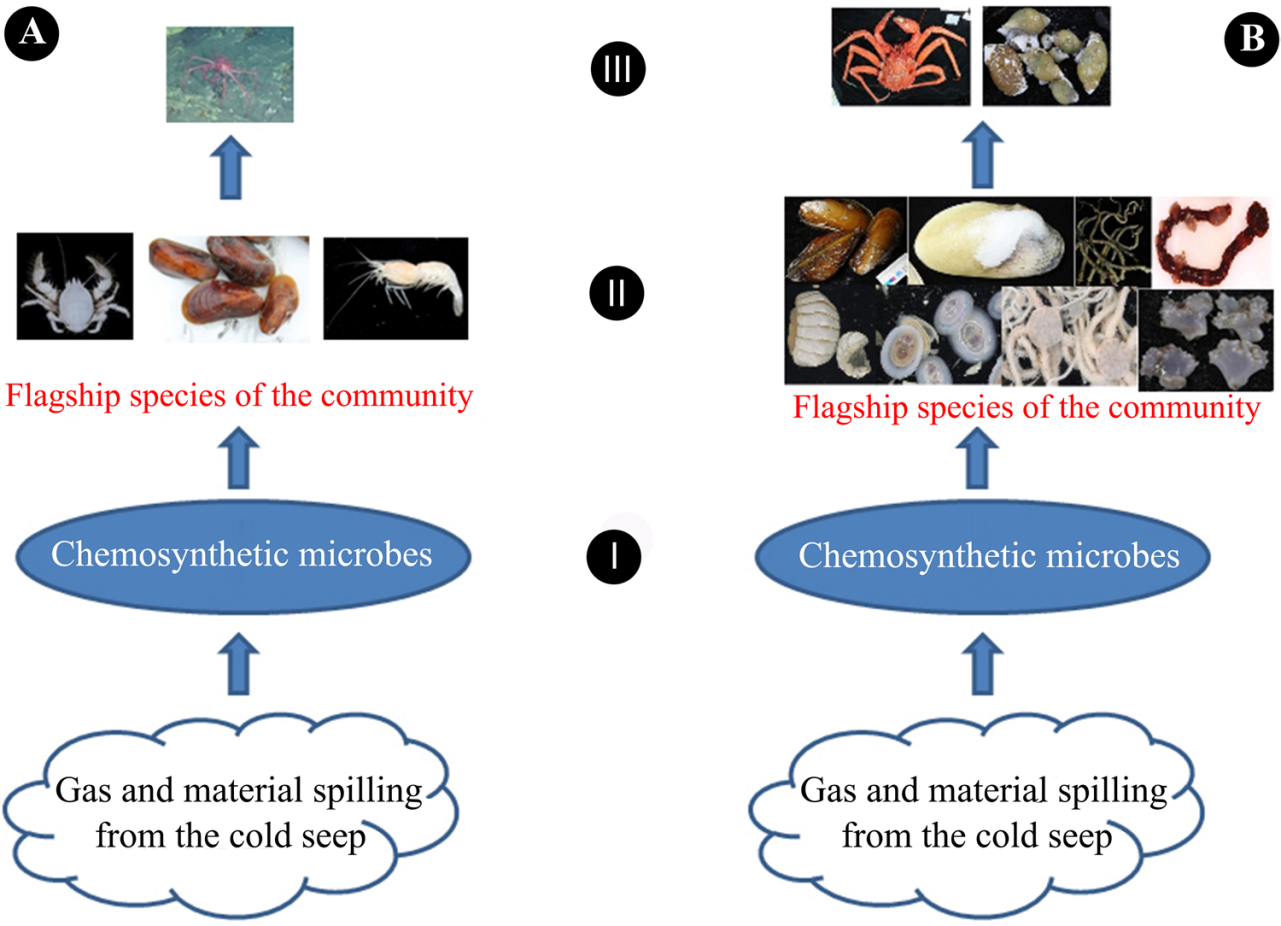
Schematic diagram of the community structures of the Site F cold-seep site (**a**) and Haima cold-seep field (**b**). **a** I, chemosynthetic microbes; II, flagship species, making up the landscape of the community, left, *Shinkaia crosnieri*, middle, *Gigantidas platifrons*, right, *Alvinocaris longirostris*; III, top predator, *Lithodes longispina* Sakai, 1971. **b** I, chemosynthetic microbes; II, flagship species, making up the landscape of the community, above, from left to right, *Gigantidas haimaensis*, *Calyptogena marissinica*, *Paraescarpia echinospica*, indeterminate species of Chiridotidae; below, from left to right, *Leptochiton tenuidontus*, *Bathyacmaea lactea*, *Histampica* sp., *Actinernus* sp.; III, top predators, *Paralomis* sp., even ?* Plicifusus* sp.

The epibenthic community structure of the Haima cold-seep field is distinct from that of Site F in the northeastern South China Sea (southwest to Taiwan Island). Site F is notable for its *Gigantidas platifrons*–*Shinkaia crosnieri* community, in which the mussel *G. platifrons* and the squat lobster *S. crosnieri* thrives in high abundance (Li 2015, 2017). The Haima cold-seep field, which is approximately 1000 km from Site F, was nearly devoid of *S. crosnieri* and has low occurrence of *G. platifrons*, with *G. haimaensis* being the dominant mussel species. Likewise, siboglinid tubeworms were common in the Haima field but scarce at Site F. The squat lobster *Munidopsis lauensis* and the alvinocaridid shrimp *Alvinocaris longirostris* were common at both cold-seep areas. The community structures at the Haima cold-seep field and the Site F are depicted in Fig. 7. At first approximation, the community structure at Haima cold seeps are roughly similar to that of chemosynthesis-based communities in Sagami Bay (Fujikura et al. 2012), although the presence of the tubeworm *Paraescarpia echinospica* links the Haima cold seeps to chemosynthetic haibitats at the Nansei-shoto (Ryukyu) Trench. In contrast, the community structure at Site F is roughly similar to that of the hydrothermal vents in the Okinawa Trough owing to the *Gigantidas platifrons*–*Shinkaia crosnieri* assemblage (Feng et al. 2018; Li 2015, 2017). The cause of such differences between cold-seep communities is still unknown. In accordance with the fieldwork experiences of the corresponding author (Xinzheng Li) at Site F in 2013 and at the Haima cold seeps in 2018, the sulfide smell of the substrate samples from the Haima cold-seep areas was much stronger than that from Site F. This phenomenon may imply that there are differences in the substrate materials, concentrations of reducing compounds, or levels of redox potentials, caused by differences in primary productivities of chemosythetic microbes. Quantitative environmental data would be needed to verify this.

Our future research will build on this preliminary taxonomic work, especially to (1) better determine community connectivity of epibenthic macroinvertebrates among different seepage sites in the South China Sea, and (2) reveal the factors that govern the community structures and ultimately determine the biodiversity at cold seeps. In conclusion, the cold seeps at both Haima and Site F merit further research, with comparisons based on more sampling and field observations, as well as comprehensive analyses that consider biological, chemical, geographic, and physical oceanographic evidence.

## Materials and methods

The investigated areas (Fig. 1) in the South China Sea are located on the northwestern continental slope and included three geomorphological environments: (1) the Haima cold-seep field, with five sites investigated, covering a depth range of 1380–1390 m; (2) a mud volcano field, at depths of 500–810 m; and (3) the slopes of Ganquan Plateau, at depths of 586–910 m on the northeastern slope, and at 1300–1412 m on the southwestern slope. At Haima cold-seep field, two sites were investigated on each dive in the manned submersible, and samples from each site were loaded together in a sampling box. Therefore, the macrofaunal specimens collected from all the five sites were examined and analysed as a single bulk sample to represent the Haima cold-seep habitat. On the southwestern slope of Ganquan Plateau, several microhabitats were present, ranging from rocky cliff to a small muddy cold-seep site in a limited area, where only siboglinid tubeworms were observed, but were integrated as part of the overall fauna of the southwestern slope.

Sampling and in situ observations were performed with the manned submersible “*Shenhai Yongshi*”. Videos and photos (Fig. 2) were taken using high-definition underwater cameras deployed on the submersible. During the cruise, only epibenthic marcoinvertebrates were qualitatively collected and analyzed. The specimens were visually detected and collected, and were therefore mostly larger than 5 mm in size. Cold-water corals, sponges, mollusks, sea urchins, and large crustaceans were directly grabbed using the mechanical arms of the submersible; small crustaceans like squat lobsters and shrimps were sampled using nets manipulated by the mechanical arms. Some adherent organisms, such as small gastropods and ophiurids, were collected together with rocks and corals. Specimens were photographed immediately after being transported to the deck of the mother vessel, and then directly frozen for conservation. Most of the specimens were retained by the research for taxonomic examination; however, specimens deposited in Tongji University and uncollected organisms observed in situ were identified only from videos and photographs.

Taxonomic studies of the specimens were mainly based on morphological method. DNA barcoding was employed to confirm the identification of some species. The samples are deposited in the Marine Biological Museum of Chinese Academy of Sciences in Qingdao, China.

## References

[CR1] Annandale N (1916). Barnacles from deep-sea telegraph cables in the Malay Archipelago. J Str Bra Roy Asi Soc.

[CR2] Baba K (1977). Five new species of chirostylid crustaceans (Decapoda, Anomura) from off Midway Island. Tokyo B Nat Sci Mus Ser A (Zool).

[CR3] Baba K, de Saint Laurent M (1992). Chirostylid and galatheid crustaceans (Decapoda: Anomura) from active thermal vent areas in the southwest. Pac Sci Mar.

[CR4] Baba K, Williams AB (1998). New Galatheoidea (Crustacea, Decapoda, Anomura) from hydrothermal systems in the West Pacific Ocean: Bismarck Archipelago and Okinawa Trough. Zoosystema.

[CR5] Baba K, Macpherson E, Lin CW, Chan TY (2009). Crustacean Fauna of Taiwan: squat lobsters (Chirostylidae and Galatheidae).

[CR6] Barry JP, Greene HG, Orange DL, Baxter CH, Robison BH, Kochevar RE, Nybakken JW, Donald LR, McHugh CM (1996). Biologic and geologic characteristics of cold seeps in Monterey Bay, California. Deep-Sea Res Pt I.

[CR7] Bourque JR, Robertson CM, Brooke S, Demopoulos AWJ (2017). Macrofaunal communities associated with chemosynthetic habitats from the U.S. Atlantic margin: A comparison among depth and habitat types. Deep-Sea Res Pt II.

[CR8] Bruce AJ (1965). On a new species of *Nephrops* (Decapoda, Reptantia) from the South China Sea. Crustaceana.

[CR9] Carney RS (1994). Consideration of the oasis analogy for chemosynthetic communities at Gulf of Mexico hydrocarbon vents. Geo-Mar Lett.

[CR10] Chan TY, Komai T (2017). A new shrimp species of the genus *Lebbeus* White, 1847 (Crustacea: Deacpoda: Caridea: Thoridae) from a deep-sea cold seep site off southwestern Taiwan. Zootaxa.

[CR11] Chen C, Okutani T, Liang Q, Qiu J (2018). A noteworthy new species of the family Vesicomyidae from the South China Sea (Bivalvia: Glossoidea). Venus.

[CR12] Cunha MR, Rodrigues CF, Génio L, Hilário A, Ravara A, Pfannkuche O (2013). Macrofaunal assemblages from mud volcanoes in the gulf of cadiz: abundance, biodiversity and diversity partitioning across spatial scales. Biogeosciences.

[CR13] Dall WH (1891). On some new or interesting west American shells obtained from the dredgings of the U.S. Fish Commission steamer Albatross in 1888, and from other sources. Proc US Nat Mus.

[CR15] Dong D, Li X (2015). Galatheid and chirostylid crustaceans (Decapoda: Anomura) from cold seep environment in the northeastern South China Sea. Zootaxa.

[CR16] D'Udekem d'Acoz C, Havermans C (2015). Contribution to the systematics of the genus *Eurythenes *SI. Smith in Scudder, 1882 (Crustacea: Amphipoda: Lysianassoidea: Eurytheneidae). Zootaxa.

[CR17] Fang Y, Wei J, Lu H, Liang J, Lu J, Fu J, Cao J (2019). Chemical and structural characteristics of gas hydrates from the Haima Cold Seeps in the Qiongdongnan Basin of the South China Sea. J Asian Earth Sci.

[CR18] Feng D, Chen D (2015). Authigenic carbonates from an active cold seep of the northern South China Sea: new insights into fluid sources and past seepage activity. Deep-Sea Res Pt II.

[CR19] Feng D, Qiu J, Hu Y, Peckmann J, Guan H, Tong H, Chen C, Chen J, Gong S, Li N, Chen D (2018). Cold seep systems in the South China Sea: An overview. J Asian Earth Sci.

[CR20] Fujikura K, Okutani T, Maruyama T (2012). Deep-Sea Life-Biological observations using research submersibles.

[CR21] Gan Z, Li X (2019). Report on four deep-water barnacles (Cirripedia, Thoracica) from the northwest Pacific, with remarks on *Trianguloscalpellum regium* (Wyville-Thomson, 1873). Zootaxa.

[CR22] German CR, Ramirez-Llodra E, Baker MC, Tyler PA, Committee CSS (2011). Deep-water chemosynthetic ecosystem research during the census of marine life decade and beyond: a proposed deep-ocean road map. PLoS ONE.

[CR23] Gong L, Li X, Qiu J (2015). Two new species of Hexactinellida (Porifera) from the South China Sea. Zootaxa.

[CR24] Hashimoto J, Okutani T (1994). Four new mytilid mussels associated with deepsea chemosynthetic communities around Japan. Venus.

[CR25] Henderson JR (1885). Diagnoses of new species of Galatheidae collected during the "Challenger" expedition. Ann Mag Nat Hist.

[CR27] Hoek PPC (1907). Pedunculata. The Cirripedia of the Siboga expedition. A. Cirripedia pedunculata. Sib-Exp Mon.

[CR29] Jiang J, Huang Y, Liang Q, Zhang J (2019). Description of two new species (Bivalvia: Vesicomyidae, Verticordiidae) from a cold seep in the South China Sea. The Nautilus.

[CR30] Kikuchi T, Ohta S (1995). Two caridean shrimps of the families Bresiliidae and Hippolytidae from a hydrothermal field on the Iheya Ridge, off the Ryukyu Islands, Japan. J Crus Biol.

[CR31] Klaucke I, Berndt C, Crutchley G, Chi WC, Lin S, Muff S (2015). Fluid venting and seepage at accretionary ridges: the Four Way Closure Ridge offshore SW Taiwan. Geo-Mar Lett.

[CR32] Kou Q, Chen J, He L, Wang Y (2017). New species of the giant deep-sea isopod genus *Bathynomus* (Crustacea, Isopoda, Cirolanidae) from Hainan Island, South China Sea. Integr Zool.

[CR33] Levin LA (2005). Ecology of cold seep sediments: interactions of fauna with flow, chemistry and microbes. Oceanogr Mar Biol Ann Rev.

[CR34] Levin LA, Mendoza GF, Grupe BM, Gonzalez JP, Jellison B, Rouse G, Thurber AR, Waren A (2015). Biodiversity on the rocks: macrofauna inhabiting authigenic carbonate at Costa Rica methane seeps. PLoS ONE.

[CR35] Li X (2015). Report on two deep-water caridean shrimp species (Crustacea: Decapoda: Caridea: Alvinocarididae, Acanthephyridae) from the northeastern South China Sea. Zootaxa.

[CR36] Li X (2017). Taxonomic research on deep-sea macrofauna in the South China Sea using the Chinese deep-sea submersible *Jiaolong*. Integr Zool.

[CR37] Liang Q, Hu Y, Feng D, Peckmann J, Chen L, Yang S, Liang J, Tao J, Chen D (2017). Authigenic carbonates from newly discovered active cold seeps on the northwestern slope of the South China Sea: Constraints on fluid sources, formation environments, and seepage dynamics. Deep-Sea Res Pt I.

[CR38] Lin CW, Osawa M, Chan TY (2007). A new *Munidopsis* (Crustacea: Decapoda: Galatheidae) associated with gorgonian corals from the deep waters off Taiwan. Proc Biol Soc Wash.

[CR39] Miura T, Hashimoto J (1991). Two new branchiate scale-worms (Polynoidae: Polychaeta) from the hydrothermal vent of the Okinawa Trough and the volcanic seamount off Chichijima Island. Proc Biol Soc Wash.

[CR40] Niu M, Fan X, Zhuang G, Liang Q, Wang F (2017). Methane-metabolizing microbial communities in sediments of the Haima cold seep area, northwest slope of the South China Sea. FEMS Microbiol Ecol.

[CR41] Okutani T, Tsuchida E, Fujikura K (1992). Five bathyal gastropods living within or near the Calyptogena community of the Hatsushima Islet, Sagami Bay. Venus.

[CR42] Paull CK, Hecker B, Commeau R, Freeman-Lynde RP, Neumann C, Corso WP, Golubic S, Hook JE, Sikes E, Curray J (1984). Biological communities at the Florida Escarpment resemble hydrothermal vent taxa. Science.

[CR43] Pilsbry HA (1907). The Barnacles (Cirripedia) contained in the collections of the U.S. National Museum B US Nat Mus.

[CR14] Pourtalès L.F. de (1878) Corals. In: Reports on the dredging operations of the U.S. Coast Survey Steamer "Blake". Bull Mus Comp Zool Harv Coll 5:197–212

[CR44] Saito H, Okutani T (1990). Two new chitons (Mollusca: Polyplacophora) from a hydrothermal vent site of the Iheya Small Ridge, Okinawa Trough, East China Sea. Venus.

[CR01] Sakai T (1971). Illustrations of 15 species of crabs of the family Lithodidae, two of which are new to science. Res Crustac.

[CR45] Sakurai K (1994). Eight new species of trochid genera, *Tristichotrochus*, *Kombologion* and *Otukaia* (Calliostomatinae) from Japan and adjacent waters. Venus.

[CR46] Sha Z (2019). Illustration of specimens collection from deep-sea
hydrothermal vents and cold seeps in Western Paciic.

[CR47] Sibuet M, Olu K (1998). Biogeography, biodiversity and fluid dependence of deep-sea cold-seep communities at active and passive margins. Deep-Sea Res Pt II.

[CR48] Southward EC, Schulze A, Tunnicliffe V (2002). *Vestimentiferans* (Pogonophora) in the Pacific and Indian Oceans: a new genus from Lihir Island (Papua New Guinea) and the Java Trench, with the first report of *Arcovestia ivanovi* from the North Fiji Basin. J Nat Hist.

[CR49] Suess E (2014). Marine cold seeps and their manifestations: geological control, biogeochemical criteria and environmental conditions. Int J Earth Sci.

[CR50] Washburn TW, Demopoulos AW, Montagna PA (2018). Macrobenthic infaunal communities associated with deep-sea hydrocarbon seeps in the northern Gulf of Mexico. Mar Eco.

[CR51] Xu T, Feng D, Tao J, Qiu J (2019). A new species of deep-sea mussel (Bivalvia: Mytilidae: *Gigantidas*) from the South China Sea: Morphology, phylogenetic position, and gill-associated microbes. Deep-Sea Res Pt I.

[CR52] Zhang S, Zhang S (2017). Description of *Pyropelta elongata* sp. nov. (Gastropoda, Pyropeltidae) from a Methane Seep Area in the South China Sea. Am Malac Bull.

[CR53] Zhang S, Zhang J, Zhang S (2016). A new species of *Bathyacmaea* (Gastropoda: Pectinodontidae) from a methane seep area in the South China Sea. The Nautilus.

